# Adult Nutrition Stress Modulates the Energy Allocation Between Migration and Reproduction in *Cnaphalocrocis medinalis*

**DOI:** 10.3390/insects17050527

**Published:** 2026-05-21

**Authors:** Chao-Min Xu, Meng-Yu Hu, Yan Wu, Ning-Ning Wu, Gao Hu, Yu-Meng Wang

**Affiliations:** 1 State Key Laboratory of Agricultural and Forestry Biosecurity, College of Plant Protection, Nanjing Agricultural University, Nanjing 210095, China; 2 Guizhou Key Laboratory of Agricultural Biosecurity, Guiyang University, Guiyang 550005, China; 3 State Key Laboratory of Agricultural and Forestry Biosecurity, College of Plant Protection, China Agricultural University, Beijing 100193, China

**Keywords:** *Cnaphalocrocis medinalis*, migration-reproduction trade-off, adult nutrition, ovarian development, flight performance

## Abstract

Insect migration is an energetically costly process often involving a trade-off with reproduction. However, how adult nutritional stress regulates resource allocation between these two life-history traits remains unclear. Here, we compared morphological traits, flight performance, ovarian development, fecundity, and energy reserves between migrants and residents of *Cnaphalocrocis medinalis* under fed and starved conditions. Morphological analyses showed no significant differences in body weight, body length or forewing length between migrants and residents, regardless of nutritional status. Under starvation, migrants exhibited significantly greater flight distances and speeds than residents, and their flight distance and duration were also higher than those of fed migrants. Starved migrants showed a higher proportion of immature ovaries and higher fecundity, accompanied by a prolonged pre-oviposition period, indicating reproductive delay. Under fed conditions, ovarian development and fecundity were similar between migrants and residents. Energy reserve assays revealed that starved migrants accumulated more abdominal triglycerides but had lower thoracic glycogen than residents, suggesting preferentially triglyceride storage in the abdomen for long-distance flight. Under fed conditions, residents possessed higher thoracic glycogen levels than migrants, whereas no differences were observed in triglyceride levels. These results indicate that *C. medinalis* prioritizes energy allocation to migration over reproduction under energy shortage, but switches to a strategy that simultaneously meets the demands of both when nutrition is sufficient. Our findings underscore the role of adult nutrition in mediating the energy allocation between migration and reproduction, offering a scientific basis for the precise monitoring and management of this pest.

## Simple Summary

Insects often face an energy allocation trade-off between migration and reproduction prior to migration. Given this trade-off, little is known about the mechanisms by which nutritional stress in the adult stage modulates resource allocation. By comparing the physiological traits of migrants and residents under varying nutritional conditions, this study elucidated the mechanism by which *Cnaphalocrocis medinalis* regulates nutrient allocation. The results showed no significant differences in morphological traits between migrants and residents, regardless of nutritional status. Under starvation conditions, migrants prioritized energy allocation to migration, as evidenced by significantly enhanced flight performance and delayed ovarian development. In contrast, when adult nutrition was supplemented, the energy demands for both migration and reproduction were simultaneously met. These findings provide new experimental evidence for elucidating the mechanisms underlying the trade-off between insect migration and reproduction.

## 1. Introduction

Every year, billions of migratory pests engage in seasonal migrations, resulting in widespread damage to agricultural crops and posing a serious threat to food security [1,2,3]. Such large-scale movements are fundamentally driven by the need to escape deteriorating environmental conditions [4]. Migratory insects typically possess both strong flight performance and high fecundity, yet both migration and reproduction are energetically costly processes that require substantial energy reserves [5,6,7]. Under limited energy reserves, a trade-off in resource allocation often occurs between migration and reproduction [8,9,10]. In the early stage of migration, insects prioritize the accumulation of energy reserves for flight, leading to the suppression of ovarian development. Following migration, the degeneration of flight muscles coincides with accelerated ovarian development [7,11,12]. This physiological antagonism between migration and reproduction is known as the “oogenesis–flight syndrome” [13,14]. The phenomenon has been observed in various insects, such as *Spodoptera frugiperda* [7], *Myzus persicae* [15], *Aphis gossypii* [16], and *Gryllus firmus* [17]. Consequently, migration in female moths usually occurs during the early stages of ovarian development [18,19,20,21].

Adult nutrition plays a critical role in regulating flight performance and reproduction in many insects [18,22,23,24], yet its effects on flight performance vary considerably across species. Starvation has been shown to impair flight performance in the oriental armyworm, *Mythimna separata* [25], and the Asian tiger mosquito, *Aedes albopictus* [26]. Notably, inadequate adult nutrition in *Agrotis ipsilon* can also inhibit flight muscle development, thereby diminishing its potential for long-distance flight [27]. However, female *A. ipsilon* fed either honey water or plain water showed no significant differences in flight performance during the first three days after emergence [28]. In contrast to its variable effects on flight, adult nutrition consistently exerts a profound influence on reproductive development [29]. Ovarian maturation and fecundity are strongly dependent on nutrient intake during the adult stage [23,30]. Starvation suppresses oocyte growth in *Romalea microptera* [31], prevents egg maturation in *Liriomyza sativae* [32], and delays ovarian development in *Macrolophus caliginosus* [33]. For most lepidopterans, ovaries are immature at emergence [30], rendering subsequent gonadal development particularly reliant on adult nutritional resources. Although it is well established that both flight performance and reproductive output are closely linked to adult nutrition in insects, how migratory species cope with variations in adult nutritional resources and further regulate the allocation of energy between flight and reproduction remains largely unexplored. Elucidating this regulatory mechanism is essential for understanding the adaptive strategies of migratory insects in heterogeneous environments.

*Cnaphalocrocis medinalis* is one of the most important migratory rice pests in China, undertaking northward migrations in spring and summer, and returning southward in autumn in response to seasonal environmental changes [34]. This species exhibits a multi-stop strategy involving dusk take-off and dawn landing, with long-distance movements potentially lasting for 3 to 5 consecutive nights. Starvation during the early adult stage increases the migration propensity of *C. medinalis* [35], whereas larval crowding or starvation does not induce migratory phenotypes but instead affects development and survival [36], highlighting that the timing of nutritional stress is critical in determining migratory outcomes. The relationship between migration and reproduction in this species appears complex. In migratory populations captured by searchlight traps, over 60% of individuals had completed ovarian development and mating, suggesting that reproductive maturation can occur during migration. Laboratory tethered flight experiments further indicated that flight activity promotes ovarian development to some extent, and mated females remain capable of subsequent migratory flights [37]. These findings point to the possibility that migration and reproduction in *C. medinalis* are not mutually exclusive, but rather exhibit a dynamic, nutrition-dependent relationship. However, the physiological mechanisms underlying how adult nutrition influences the trade-off between migration and reproduction remain poorly understood.

Migratory moths are often morphologically indistinguishable from non-migratory individuals. However, both field and laboratory observations have revealed that migratory individuals of *C. medinalis* typically take off in a vertical spiral pattern at dusk [38]. In this study, we used this behavioral assay under simulated dusk conditions to categorize them as migratory (MG) or non-migratory (NMG) individuals, and subsequently assessed morphological differences between the two groups by measuring body weight, body length, and forewing length under different nutritional conditions. Furthermore, to elucidate how adult nutrition modulates the migration–reproduction trade-off, we assessed flight performance via tethered flight experiments, compared ovarian development and fecundity, and quantified triglyceride and glycogen reserves in the thorax and abdomen. These findings provide insights into the physiological mechanisms underlying the trade-off between migration and reproduction.

## 2. Materials and Methods

### 2.1. Insect Rearing

*C. medinalis* individuals were collected in September 2010 from rice fields at Jiangpu Farm and the Academy of Agricultural Sciences in Nanjing, China. The population was established following the method described previously [39]. All insects were reared in artificial incubators (Model PRX-450C, Ningbo Saifu Experimental Instrument Co., Ltd., Ningbo, China) under controlled environmental conditions (27 ± 1 °C, photoperiod L:D = 14:10) for over sixty generations. Larvae were reared on wheat seedlings, and pupae were transferred to plastic cups (1000 mL) containing moistened cotton to maintain humidity. Newly emerged female adults were placed in transparent plastic cups (330 mL). For the Feeding treatment, females were continuously provided with cotton balls soaked in 5% honey solution. For the starvation treatment, adults were given only water-moistened cotton balls on the 1st day after emergence, and subsequently fed with 5% honey solution from the night of day 1 (Table 1). Each cup contained five adults to prevent overcrowding.

### 2.2. Evaluation of Migratory Behavior

To distinguish between MG and NMG individuals of *C. medinalis*, a migratory behavior observation was performed. Female moths were placed in cylindrical cages 1 h prior to the experiment for acclimation to the test conditions (27 ± 0.3 °C, 50 ± 5% RH). Each cage was constructed from transparent polyvinyl chloride (PVC), measuring 60 cm in diameter and 120 cm in height. To simulate twilight conditions, 10 adjustable full-spectrum lights were installed, and the light intensity was gradually reduced from 1000 lx to 0.1 lx within 30 min. Migratory behavior was observed from outside the transparent cage with a red-light flashlight during the 30 min after the end of the twilight period. Moths that took off in a vertical spiral pattern and ascended to the top of the cage were classified as MG individuals, whereas those that flew randomly without reaching the top or remained stationary were designated as NMG individuals [40]. Both MG and NMG individuals were subsequently collected for further analyses.

### 2.3. Flight Performance Assessment

Following behavioral observation, flight performance was measured using an 8-channel infrared insect flight mill (China Patent No. ZL202121642579.3) based on the Stm32f103 microcontroller [41]. Each moth was placed in a test tube and chilled on ice for approximately 2 min until anaesthetized. A brush was used to gently remove scales from the junction of the thorax and abdomen. A capillary glass tube was then dipped lightly into nail-free glue and attached to the tergum. Subsequently, the tip of the flight arm was coated with a small amount of cyanoacrylate glue (Pattex; Henkel, Düsseldorf, Germany) and affixed to the insect. The flight arm was then re-installed on the flight mill. After recovering from cold immobilization, the moth began to rotate the arm, and the number of rotations was recorded at 5 s intervals. Flight distance and speed were calculated based on the rotation counts and the diameter of the flight arm, either for the entire experimental period or per unit time. All tests were conducted in complete darkness at 27 °C and 70% RH until the moth died.

### 2.4. Fecundity Assay

Reproductive performance of *C. medinalis* was assessed by measuring pre-oviposition period, number of eggs per female, oviposition duration, mating rate, mating frequency and adult longevity. MG and NMG individuals were paired at a 1:1 ratio and placed in 360 mL transparent plastic cups sealed with plastic wrap. Fresh cups and cotton balls soaked in 5% honey solution were provided daily. The age at first oviposition was recorded for each female. Eggs deposited on the plastic wrap and inner cup walls were counted daily until female death, and total number of eggs and oviposition duration were subsequently calculated. Following death, the female abdomen was dissected to examine the bursa copulatrix for the presence of spermatophores. The presence of at least one spermatophore indicated successful mating, and the number of spermatophores was recorded as mating frequency. Mating rate was calculated as the proportion of mated females relative to the total number of females tested.

### 2.5. Ovarian Developmental Assay

Ovarian development was compared between MG and NMG individuals, under both fed and starved conditions following migratory behavior observation. Fed moths were maintained individually with 5% honey solution in an unmated state following the migratory observation, and ovarian dissections were performed daily at a fixed time from day 2 to day 5. Ovarian maturation was classified into ten grades [42], based on yolk deposition, number of mature eggs, and fat body morphology. Grade I: ovarioles are short, soft and transparent, lacking discernible egg cells. Grade II: ovarioles remain largely transparent, with faint outlines of immature oocytes. Grade III: ovarioles are transparent, with visible transparent egg cells. Grade IV: milky white yolk occupies approximately 1/3 of the egg at the base of the ovariole. Grade V: milky white yolk occupies approximately 1/2 of the egg at the base of the ovariole. Grade VI: milky white yolk occupies approximately 2/3 of the egg at the base of the ovariole. Grade VII: 1–2 pale yellow mature eggs are visible at the base of the ovariole. Grade VIII: 3–5 pale yellow mature eggs are visible at the base of the ovariole. Grade IX: 6–10 pale yellow mature eggs are visible at the base of the ovariole. Grade X: approximately 10–15 mature eggs are present at the base of the ovariole. Eggs are present in the oviduct, and most of the fat bodies exhibit a filamentous morphology.

### 2.6. Determination of Triglyceride and Glycogen Contents

To compare the energy storage between MG and NMG individuals of *C. medinalis* under fed and starved conditions, triglycerides and glycogen were measured separately in the thorax and abdomen of female moths. Tissues from every 5 individuals were pooled to obtain sufficient material for detection.

Triglyceride content was measured using a commercial assay kit (Nanjing Jiancheng Bioengineering Institute, Nanjing, China; Cat. No. A110-1-1). Thoracic and abdominal tissues were homogenized in 100 μL of ice-cold normal saline, separately. The homogenate was then centrifuged at 2500 rpm for 10 min at 4 °C. A 2.5 μL aliquot of the supernatant was mixed with 250 μL of assay buffer and incubated at 37 °C for 10 min. Absorbance was measured at a wavelength of 500 nm using a SpectraMax M5 (Molecular Devices, LLC, San Jose, CA, USA) microplate reader. Triglyceride content was calculated based on the protein content of the corresponding sample.

Glycogen content was determined using a glycogen assay kit (Comin, Suzhou, China; Cat. No. TY-1-Y). Tissues were thoroughly homogenized in the extraction solution (0.1 g tissue per 750 μL solution), separately. The homogenate was heated in a 95 °C water bath for 20 min, with vortex every 5 min to ensure complete dissolution. After cooling, the volume was adjusted to 5 mL with distilled water. The solution was mixed thoroughly and centrifuged at 8000× *g* for 10 min at 25 °C. A 60 μL aliquot of the supernatant was mixed with 240 μL assay buffer and incubated in a 95 °C water bath for 10 min. The absorbance was measured at 620 nm. Glycogen content was calculated based on the protein content of each sample.

Protein content was determined in parallel using a total protein assay kit (Nanjing Jiancheng Bioengineering Institute, Nanjing, China; Cat. No. A045-1-2). An aliquot of 10 μL supernatant was mixed with 250 μL of assay buffer and incubated at 37 °C for 30 min. Absorbance was measured at 562 nm using a SpectraMax M5 microplate reader.

### 2.7. Statistical Analysis

Morphological parameters, which were normally distributed, were analyzed using Student’s *t* test. Migratory rate, proportion of individuals with immature ovaries, and mating rate were compared between groups using the Chi-square test. The data for flight performance and fecundity parameters did not follow the normal distribution. These parameters were analyzed using the Mann-Whitney U test. In addition, a two-way ANOVA for trimmed means was applied to examine interaction effects, with Q-values and *p*-values returned by R (v4.1.3, https://www.r-project.org/, accessed on 19 March 2026). When the interaction was significant, post-hoc multiple comparisons were conducted using Dunn’s test with Bonferroni correction. Energy substrate contents were evaluated by one-way ANOVA. A two-way ANOVA was conducted to examine the interactive effects of nutritional condition and migratory behavior on morphological parameters and energy substrate contents. All statistical analyses were performed using IBM SPSS Statistics 25.0. Data are presented as mean ± SD. All figures were generated using GraphPad Prism 8.0.1.

## 3. Results

### 3.1. Starved MG Individuals Exhibit Enhanced Flight Performance Compared with NMG Individuals

**Figure 1:**
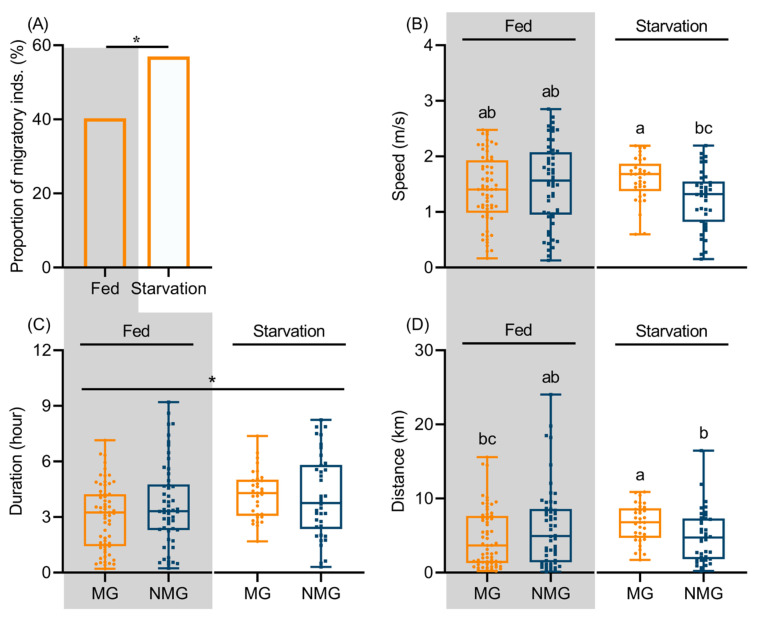
Migratory proportion (**A**), flight speed (**B**), duration (**C**) and distance (**D**) of *C. medinalis* under different nutritional conditions. Solid bars represent medians, boxes represent the interquartile range, whiskers extend to observations within ± 1.5 times the interquartile range, * *p* < 0.05 The asterisk in panel C indicates a significant main effect of nutritional condition on flight duration. MG and NMG denotes migratory and non-migratory individuals respectively. Gray-shaded and white, unshaded backgrounds represent the fed and starved groups, respectively. Different lowercase letters indicate significant differences among different treatments.

Under starved conditions, the proportion of MG individuals of *C. medinalis* reached approximately 57% (53/93), which was significantly higher than the 40.28% (29/72) observed under fed conditions (χ^2^ = 4.534, df = 1, *p* = 0.033; Figure 1A). Comparisons of morphological traits between MG and NMG individuals under different nutritional conditions revealed that neither nutritional condition nor migratory status exerted a significant effect on any morphological parameter (all *p* > 0.05; Tables S1 and S2).

To investigate the effects of nutritional condition and migratory status on flight performance of *C. medinalis*, we compared flight parameters between MG and NMG individuals under different nutritional conditions (Table S3). There was a significant interaction between nutritional condition and migratory status for flight speed (*p* = 0.010) and flight distance (*p* = 0.007), but not for flight duration (*p* = 0.348). Nutritional condition significantly increased flight duration (*p* = 0.011), whereas migratory status had no significant effect on flight duration (Table S4). Under both fed and starved conditions, no significant differences in flight duration were observed between MG and NMG individuals (*p* > 0.05) Pairwise comparisons further revealed that under starvation, MG individuals exhibited significantly greater flight speed than NMG individuals (*p* = 0.003, Z = −3.009). For flight distance, starved MG individuals outperformed both starved NMG individuals (*p* = 0.007, Z = −2.698) and fed MG individuals (*p* = 0.002, Z = −3.174). All remaining pairwise comparisons were not statistically significant (all *p* > 0.05, Figure 1B–D). Thus, the interaction between nutritional condition and migratory status critically mediates the starvation-induced enhancement of flight speed and distance, whereas flight duration is prolonged solely by nutritional stress, independent of migratory status. Collectively, these results demonstrate that starvation promotes flight performance in MG individuals.

### 3.2. Starved MG Individuals Show Delayed Reproduction

**Figure 2:**
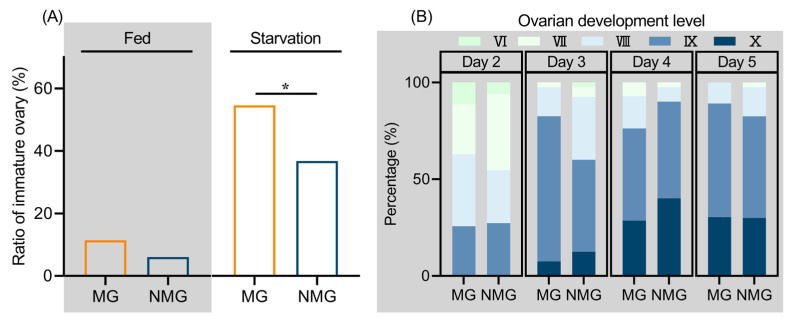
Proportion of individuals with immature ovaries in two-day-old adults under fed and starvation conditions (**A**), and ovarian grades of MG and NMG individuals from day 2 to day 5 after emergence under fed conditions (**B**). * *p* < 0.05. MG and NMG denotes migratory and non-migratory individuals respectively. Gray-shaded and white, unshaded backgrounds represent the fed and starved groups, respectively.

To explore the trade-off between migration and ovarian development of *C. medinalis,* we assessed ovarian development in MG and NMG adults following migratory behavior experiments (Table S5). Under starvation, the majority of individuals exhibited immature ovaries (stage I–VI), with a minority reaching maturity (stage VII). The proportion of MG individuals with immature ovaries (54.55%) was significantly higher than that of NMG individuals (36.76%, *p* = 0.039), indicating a delay in ovarian maturation under nutrient stress. In contrast, under fed conditions, most females had ovaries at stage VII or above, with mature eggs visible at the ovariole base. Although the proportion with immature ovaries was slightly higher in MG (11.43%) than in NMG (6.06%) individuals, the difference was not statistically significant (*p* = 0.435; Figure 2A). Ovarian development was also compared across ages (Table S6). While the number of mature eggs began to increase from 3 days post-emergence onward, no significant differences in ovarian level were detected between MG and NMG individuals at 2, 3, 4, or 5 days after emergence (Figure 2B), indicating that the progression of ovarian maturation is similar between the two groups under adequate nutrition.

**Figure 3:**
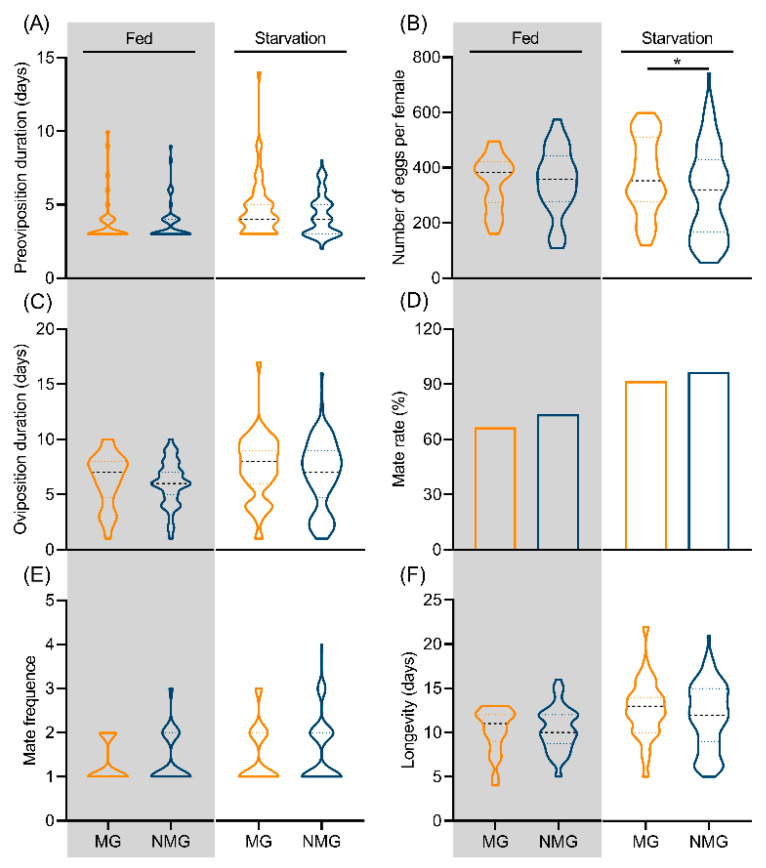
Reproductive parameters of MG and NMG moth under different nutritional conditions. Pre-oviposition period (**A**), egg production (**B**), oviposition duration (**C**), mating rate (**D**), mating frequency (**E**), and longevity (**F**). * *p* < 0.05. MG and NMG denotes migratory and non-migratory individuals respectively. Gray-shaded and white, unshaded backgrounds represent the fed and starved groups, respectively.

To clarify the roles of nutritional condition and migratory status in the fecundity of *C. medinalis*, we first analyzed their interaction and detected no significant interaction effect (Tables S7 and S8). However, nutritional condition alone significantly prolonged the pre-oviposition period (*p* = 0.001), oviposition duration (*p* = 0.037), and adult longevity (*p* = 0.001), whereas migratory status alone had no significant effect on any reproductive trait. We then compared fecundity traits between MG and NMG adults under both starvation and fed conditions. Following nutritional restriction, starved MG individuals laid significantly more eggs than NMG individuals (*p* = 0.027, Z = −2.213). Although MG individuals exhibited a slightly longer pre-oviposition period (*p* = 0.180, Z = −1.340), oviposition duration (*p* = 0.303, Z = −1.030), and adult longevity (*p* = 0.312, Z = −1.011) than NMG individuals, these differences were not statistically significant (all p > 0.05). Moreover, no significant differences were found in mating rate (χ^2^ = 2.029, *p* = 0.154) or mating frequency (*p* = 0.374, Z = −0.888). Under fed conditions, no significant differences were observed between MG and NMG in any fecundity related traits (all *p* > 0.05; Figure 3). Collectively, under starvation, MG individuals exhibit delayed reproductive development, whereas under favorable nutrition, reproductive traits are comparable between the two phenotypes.

### 3.3. Starved Migratory Individuals Prioritize Energy Allocation to Migration

**Figure 4:**
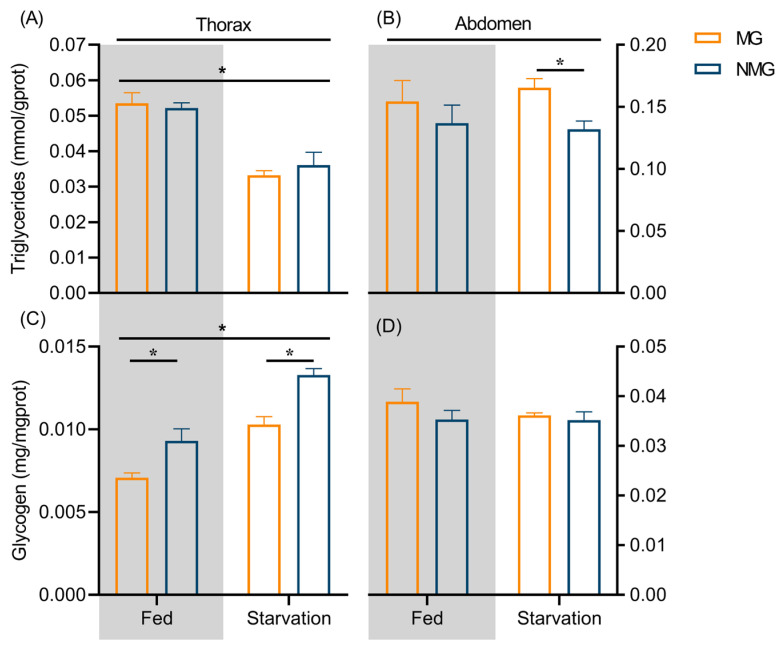
Comparison of energy substrates in the thorax and abdomen of MG and NMG female moth under different nutritional conditions. (**A**) and (**B**) represent triglyceride contents in thorax and abdomen respectively. (**C**) and (**D**) represent glycogen contents in thorax and abdomen respectively. * *p* < 0.05. The asterisk in panel (**A**) indicates a significant main effect of nutritional condition on thoracic triglyceride content. The asterisk above panel (**C**) indicates a significant main effect of nutritional condition on thoracic glycogen content, and the lower asterisk in panel (**C**) and panel (**B**) represents a significant difference in thoracic glycogen content between MG and NMG individuals. MG and NMG denotes migratory and non-migratory individuals respectively. Gray-shaded and white, unshaded backgrounds represent the fed and starved group, respectively.

To elucidate the relationship between migratory behavior and energy reserves in *C. medinalis*, we measured triglyceride and glycogen contents in thorax and abdomen of MG and NMG moths under different nutritional conditions (Table S9). No significant interaction between the nutritional conditions and migratory status was detected for energy substrate (Table S10). Regarding main effects, nutritional conditions significantly affected both thoracic triglyceride and glycogen levels. Specifically, starvation decreased thoracic triglyceride (*p* < 0.001) but increase thoracic glycogen (*p* < 0.001) compared to fed conditions. Migratory status alone was associated with a decrease in thoracic glycogen (*p* = 0.001, F = 28.2) and a potential increase in abdominal triglyceride, although the latter fell just short of significance (*p* = 0.071, F = 4.349; the interaction was pooled with error, *p* = 0.059, F = 4.648). To further characterize these differences, we found that under both fed and starved conditions, NMG individuals exhibited significantly higher thoracic glycogen levels than MG individuals (fed: *p* = 0.045, F = 8.261; starved: *p* = 0.008 F = 24.560). Under starvation, abdominal triglyceride levels were significantly higher in MG than in NMG individuals (*p* = 0.028, F = 11.331), whereas thoracic triglyceride and abdominal glycogen did not differ between the two groups. In contrast, no significant differences were detected between MG and NMG in thoracic and abdominal triglyceride, abdominal glycogen under fed conditions (all *p* > 0.05, Figure 4). Taken together, these findings demonstrate that migratory status is associated with reduced thoracic glycogen stores, suggesting that this reduction may reflect glycogen utilization as an energy source during the initial phase of migration. Furthermore, the elevated abdominal triglycerides observed in starved MG individuals support the notion of a strategic energy reallocation to fuel migratory flight under nutritional stress.

## 4. Discussion

Migratory insects often face an energetic trade-off between migration and reproduction prior to emigration, owing to the finite allocation of internal energy resources. The temporary suppression of ovarian development serves as a physiological strategy to meet the high energetic demands of migration flight [8,12,19]. In this study, we investigated the physiological mechanisms underlying energy allocation between migration and reproduction under different nutritional conditions in the rice leaf roller, *C. medinalis*. Our results demonstrate that migratory individuals experiencing nutritional stress in the early adult stage prioritize energy allocation for flight, whereas adequate nutritional resources enable them to meet the demands of both migration and reproduction concurrently. Overall, these findings advance our understanding of the adaptive strategies of *C. medinalis* in heterogeneous environments and provide a theoretical foundation for integrated pest management of this species.

Take-off behavior represents the initial and critical step in insect migration, playing an essential role in determining whether an individual successfully initiates migration [43,44]. Field observations have documented that migratory individuals of *C. medinalis* typically take off in a vertical spiral pattern, which is distinctly different from the horizontal or irregular short-distance flights such as foraging or mate seeking [38]. Comparative behavioral assays conducted on field-collected emigrating and local resident populations revealed that the proportion of individuals exhibiting migratory take-off behavior was significantly higher in emigrating populations than in resident populations. These findings support the applicability of take-off behavior as a reliable indicator of migratory propensity in insects, an approach that has also been validated in *Laodelphax striatellus*, *Oedaleus asiaticus*, *S. frugiperda* [45,46,47]. Using this method to distinguish between migrants and residents, we further compared their morphological and physiological traits under different nutritional conditions. The results showed that under starvation conditions, migratory individuals of *C. medinalis* exhibited delayed ovarian development and enhanced flight speed and flight distance compared to residents. However, under fed conditions, no significant differences in these traits were detected between the two phenotypes, even though 40.28% of the population exhibited migratory behavior. Further analysis revealed that flight performance was interactively affected by nutritional condition and migratory status. Specifically, the combination of starvation and migratory tendency synergistically enhanced flight speed and flight distance but not flight duration, whereas under fed conditions, this interactive effect diminished. These findings indicate that ovarian development and flight performance are dynamic traits modulated by adult nutritional condition, and that reliance on these physiological indicators alone may underestimate migratory propensity under favorable nutritional conditions.

The larval stage is widely recognized as a critical period for nutrient acquisition and environmental response in most lepidopteran insects. Consequently, extensive research has focused on how larval nutrition influences migratory behavior, with studies in species such as *M. separata* [48], *S. frugiperda* [47,49], *Loxostege sticticalis* [50], and *C. medinalis* [36]. In contrast, the role of adult nutrition in regulating migration has received far less attention, although emerging evidence suggests it is equally important. For example, inadequate nutrition on the day of flight significantly reduces flight duration in *Locusta migratoria* [51], and in *M. separata*, starvation within 24 h after emergence triggers a shift from migrants to residents, characterized by reduced flight performance [52]. These findings indicate that for many species, adult nutritional stress suppresses migratory potential. However, our study reveals a contrasting pattern in *C. medinalis*: starvation during the adult stage enhances migratory propensity, leading to longer flight distances and faster flight speeds. This divergence may reflect species-specific adaptations to ecological pressures. In particular, this response aligns with the ecological context of its autumn southward migration, during which rice plants senesce, host plant nutritional quality declines, and nectar sources for adults become scarce [53]. Thus, for *C. medinalis*, adult starvation may represent an environmental cue rather than a physiological constraint, promoting adaptive migration from deteriorating habitats. This adaptive strategy may enhance population persistence in temporally and spatially heterogeneous environments.

A trade-off between migration and reproduction, often referred to as the “oogenesis-flight syndrome,” has been widely documented in insects [54]. This phenomenon has been observed in species such as *M. separata* [52], *S. frugiperda* [7], *Spodoptera exempta* [55] and *Nilaparvata lugens* [56], although it appears to be absent in *Spodoptera exigua* [57]. The essence of this trade-off lies in the limitation of energy reserves, raising the question of whether it persists under resource-rich conditions. In *M. separata*, nutrient deprivation suppresses flight muscle development and accelerates reproductive maturation only when experienced during the first 24 h after emergence, triggering a shift from migrants to residents [52]. Similarly, this study demonstrates that in *C. medinalis*, the migration–reproduction trade-off is nutrition-dependent. Under nutrient deficiency, individuals prioritize energy allocation to migration at the expense of reproduction, resulting in delayed ovarian development. Conversely, when nutritional resources are sufficient, both migratory and reproductive demands are met concurrently, and no apparent trade-off is observed. These findings indicate that the trade-off is not an intrinsic trait but is dynamically modulated by adult nutritional conditions. The role of adult nutrition in modulating this trade-off in other migratory insects deserves further investigation.

Prior to migration, insects typically undergo physiological preparation for prolonged flight. The primary energy substrates fueling migration are derived from the fat body [5], with glycogen and triglycerides serving as key storage forms [6]. During flight, energy metabolism exhibits a temporal transition in which glycogen is predominantly utilized during the initial phase, whereas triglycerides become the primary fuel source to sustain long-duration flight [6,58]. The allocation of energy reserves associated with migratory behavior varies considerably across species. Migratory populations of *Plutella xylostella* exhibited significantly higher triglyceride content in the thorax compared to residents, whereas no significant difference was detected in the abdomen [59]. In S. frugiperda, migratory individuals accumulate higher abdominal triglyceride levels than their resident counterparts [7]. In our study, no significant interaction between nutritional condition and migratory status was detected for energy substrate, indicating that the two factors independently regulate energy reserves in *C. medinalis*. Under fed conditions, no significant differences in triglyceride content were detected between migratory and resident individuals in either body segment. However, under starvation, migratory individuals accumulated significantly more abdominal triglycerides, indicating that *C. medinalis* prioritizes energy allocation to migration under nutritional stress. Notably, thoracic energy substrates (both glycogen and triglycerides) were highly sensitive to nutritional condition, whereas abdominal energy reserves remained largely unaffected by starvation. This differential sensitivity implies that the thorax serves as a dynamic energy hub that responds rapidly to changes in food availability, while the abdomen functions primarily as a long-term storage depot. Interestingly, thoracic glycogen levels were consistently higher in residents than in migrants under both nutritional conditions, aligning with findings in *S. frugiperda* [7]. We speculate that lower thoracic glycogen in migrants reflects depletion due to take off activity, given glycogen’s role as the primary fuel for flight initiation. Taken together, these results suggest that in response to nutritional stress, *C. medinalis* first mobilizes thoracic energy substrates to ensure successful energy supply during the early phase of migration. This energy allocation strategy may enhance migratory endurance and survival in nutritionally poor environments, thereby facilitating long-distance dispersal and colonization of ephemeral habitats.

## Figures and Tables

**Table 1:** Different treatment schemes of *C. medinalis* included in the study.

Treatment	Adult Age (Day)	
	1st	2nd
Fed		
Starvation		

Note: Black bars represent fed with 5% honey solution and white bars represent fed with water.

## Data Availability

The original contributions presented in this study are included in the article. Further inquiries can be directed to the corresponding author (yumengwang@njau.edu.cn).

## Supplementary Materials

The following supporting information can be downloaded at: https://www.mdpi.com/article/10.3390/insects17050527/s1, Table S1: Two-way ANOVA for the effects of nutritional conditions, migratory status on the morphological characteristics in migratory (MG) and non-migratory (NMG) individuals of *C. medinalis*; Table S2: Morphological characteristics in migratory (MG) and non-migratory (NMG) individuals of *C. medinalis* under different nutritional conditions; Table S3: Flight parameters in migratory (MG) and non-migratory (NMG) individuals of *C. medinalis* under different nutritional conditions; Table S4: Two-way ANOVA for the effects of nutritional conditions and migratory status on the flight capability in migratory (MG) and non-migratory (NMG) individuals of *C. medinalis;* Table S5: The proportion of immature ovaries in migratory (MG) and non-migratory (NMG) individuals of *C. medinalis* at 2nd days under different nutritional conditions; Table S6: Ovarian development grades in migratory (MG) and non-migratory (NMG) individuals of *C. medinalis* at 2nd to 5th days under fed conditions; Table S7: Reproductive parameters in migratory (MG) and non-migratory (NMG) individuals of *C. medinalis* under different nutritional conditions; Table S8: Two-way ANOVA for the effects of nutritional conditions and migratory status on the reproductive parameters in migratory (MG) and non-migratory (NMG) individuals of *C. medinalis;* Table S9: The content of energy substances in migratory (MG) and non-migratory (NMG) individuals of *C. medinalis* under different nutritional conditions; Table S10: Two-way ANOVA for the effects of nutritional conditions, migratory status on the content of thoracic and abdominal energy substances in migratory (MG) and non-migratory (NMG) individuals of *C. medinalis*.
