# Supplementary material for: Adult Nutrition Stress Modulates the Energy Allocation Between Migration and Reproduction in Cnaphalocrocis medinalis
Source: Insects. 2026 May 21;17(5):527. doi: 10.3390/insects17050527 (PMC13207850; doi:10.3390/insects17050527)
Supplement: Supplementary file 1 [file insects-17-00527-s001.zip › insects-4237932-supplementary.pdf]

Supplementary information

Adult nutrition stress modulates the energy allocation between migration and reproduction in *Cnaphalocrocis medinalis*

Chao-Min Xu [1], Meng-Yu Hu [1], Yan Wu [2], Ning-Ning Wu [3], Gao Hu [1,2,*] and Yu-Meng Wang [1,*]

[1]*State Key Laboratory of Agricultural and Forestry Biosecurity, College of Plant Protection, Nanjing Agricultural University, Nanjing 210095, China*

[2]*Guizhou Key Laboratory of Agricultural Biosecurity, Guiyang University, Guiyang 550005, China*

[3] *State Key Laboratory of Agricultural and Forestry Biosecurity, College of Plant Protection, China Agricultural University, Beijing 100193, China*

* **Correspondence** Gao Hu (hugao@njau.edu.cn), Yu-Meng Wang (yumengwang@njau.edu.cn)

Table S1: Two-way ANOVA for the effects of nutritional conditions, migratory status on the morphological characteristics in migratory (MG) and non-migratory (NMG) individuals of *C. medinalis*.

Parameters	Source	df	MS	*F*	*p*
Weight (mg)	Fed vs Starvation	1	1.03	0.157	0.692
	MG vs NMG	1	0.156	0.024	0.878
	Fed vs Starvation × MG vs NMG	1	0.048	0.007	0.932
	Error	150	6.549		
Body length (mm)	Fed vs Starvation	1	0.259	0.527	0.469
	MG vs NMG	1	0.014	0.028	0.867
	Fed vs Starvation × MG vs NMG	1	0.005	0.011	0.916
	Error	150	0.492		
Forewing length (mm)	Fed vs Starvation	1	0.192	0.404	0.526
	MG vs NMG	1	0.001	0.002	0.969
	Fed vs Starvation × MG vs NMG	1	0.016	0.033	0.855
	Error	150	0.476		

Table S2: Morphological characteristics in migratory (MG) and non-migratory (NMG) individuals of *C. medinalis* under different nutritional conditions.

Morphological parameters		n (MG/NMG)	MG	NMG
Fed	Body length (mm)	39/35	8.55 ± 0.71	8.55 ± 0.70
	Forewing length (mm)	39/35	8.37 ± 0.73	8.40 ± 0.60
	Weight (mg)	39/35	14.64 ± 2.58	14.54 ± 2.45
Starvation	Body length (mm)	42/38	8.47 ± 0.68	8.46 ± 0.73
	Forewing length (mm)	42/38	8.32 ± 0.68	8.30 ± 0.73
	Weight (mg)	42/38	14.77 ± 2.76	14.74 ± 2.41

Data are presented as mean ± SD.

Table S3: Flight parameters in migratory (MG) and non-migratory (NMG) individuals of *C. medinalis* under different nutritional conditions.

Flight parameters		Statistical analysis parameters		
		n (MG/NMG)	MG	NMG
Fed	Flight speed (km/h)	63/54	1.40 ± 0.62	1.51 ± 0.73
	Flight duration (h)	63/54	3.04 ± 1.77	3.66 ± 2.26
	Flight distance (km)	63/54	4.63 ± 3.94	6.08 ± 5.54
Starvation	Flight speed (km/h)	33/38	1.59 ± 0.40	1.23 ± 0.53
	Flight duration (h)	33/38	4.22 ± 1.29	4.12 ± 2.24
	Flight distance (km)	33/38	6.70 ± 2.52	4.94 ± 3.54

Data are presented as mean ± SD.

Table S4: Two-way ANOVA for the effects of nutritional conditions and migratory status on the flight capability in migratory (MG) and non-migratory (NMG) individuals of *C. medinalis*.

Parameters	Source	Q-value	*p*-value
Flight speed (km/h)	Fed vs Starvation	0.1371	0.712
	MG vs NMG	1.7020	0.195
	Fed vs Starvation × MG vs NMG	6.9004	0.010
Flight duration (h)	Fed vs Starvation	6.8102	0.011
	MG vs NMG	0.1444	0.705
	Fed vs Starvation × MG vs NMG	0.8938	0.348
Flight distance (km)	Fed vs Starvation	3.2571	0.074
	MG vs NMG	0.7782	0.380
	Fed vs Starvation × MG vs NMG	7.6458	0.007

Data of these parameters didn't fit the normal distribution, thus the two-way ANOVA for trimmed means with interactions effects was applied, and Q-value and *p*-value were returned.

Table S5: The proportion of immature ovaries in migratory (MG) and non-migratory (NMG) individuals of *C. medinalis* at 2nd days under different nutritional conditions

Treatment		Statistical analysis parameters				
		n	percentage*	χ^2	df	*p*
Fed	MG	35	11.43%	0.608	1	0.435
	NMG	33	6.06%			
Starvation	MG	66	54.55%	4.27	1	0.039
	NMG	68	36.76%			

*The percentages in the table refer to the proportion of immature ovaries to the total number of ovarian samples. The data were analyzed using the Chi-square test.

Table S6: Ovarian development grades in migratory (MG) and non-migratory (NMG) individuals of *C. medinalis* at 2nd to 5th days under fed conditions.

Adult age	Ovarian development grades	MG (percentage)*	NMG (percentage)*
2	VI	4 (11.43%)	2 (6.06%)
	VII	9 (25.71%)	13 (39.39%)
	VIII	13 (37.14%)	9 (27.27%)
	IX	9 (25.71%)	9 (27.27%)
	X	0 (0%)	0 (0%)
	Total	35	33
3	VI	0 (0%)	1 (2.5%)
	VII	1 (2.5%)	2 (5%)
	VIII	6 (15%)	13 (32.5%)
	IX	30 (75%)	19 (47.5%)
	X	3 (7.5%)	5 (12.5%)
	Total	40	40
4	VI	0 (0%)	0 (0%)
	VII	3 (7.14%)	1 (2.5%)
	VIII	7 (16.67%)	3 (7.5%)
	IX	20 (47.62%)	20 (50%)
	X	12 (28.57%)	16 (40%)
	Total	42	40
5	VI	0 (0%)	0 (0%)
	VII	0 (0%)	1 (2.5%)
	VIII	5 (10.87%)	6 (15%)
	IX	27 (58.7%)	21 (52.5%)
	X	14 (30.43%)	12 (30%)
	Total	46	40

*The percentages in parentheses correspond to the proportions of each ovarian grade.

Table S7: Reproductive parameters in migratory (MG) and non-migratory (NMG) individuals of *C. medinalis* under different nutritional conditions.

Treatment		Pre-oviposition duration (n*)	Egg production (n*)	Oviposition duration (n*)	Mating frequency (n*)	Longevity (n*)
Fed	MG	3.97 ± 1.79 (30)	346.04 ± 98.64 (23)	6.33 ± 2.40 (30)	1.29 ± 0.46 (31)	10.27 ± 2.52 (48)
	NMG	3.79 ± 1.37 (42)	351.00 ± 120.34 (28)	6.02 ± 2.03 (43)	1.38 ± 0.58 (42)	10.33 ± 2.48 (54)
Starvation	MG	4.75 ± 2.03 (51)	372.24 ± 137.95 (46)	7.45 ± 2.90 (51)	1.46 ± 0.66 (56)	12.66 ± 3.31 (61)
	NMG	4.24 ± 1.39 (90)	309.08 ± 163.74 (85)	6.84 ± 3.15 (90)	1.58 ± 0.73 (92)	11.88 ± 3.69 (97)

*Values in parentheses denote the sample size. Data are presented as mean ± SD.

Table S8: Two-way ANOVA for the effects of nutritional conditions and migratory status on the reproductive parameters in migratory (MG) and non-migratory (NMG) individuals of *C. medinalis*.

Parameters	Source	Q-value	*p*-value
Pre-oviposition duration (days)	Fed vs Starvation	18.9330	0.001
	MG vs NMG	0.7563	0.387
	Fed vs Starvation × MG vs NMG	0.8846	0.350
Egg production	Fed vs Starvation	0.6583	0.420
	MG vs NMG	2.0004	0.161
	Fed vs Starvation × MG vs NMG	2.5558	0.114
Oviposition duration (days)	Fed vs Starvation	4.5229	0.037
	MG vs NMG	1.8131	0.182
	Fed vs Starvation × MG vs NMG	0.0001	0.994
Mating frequency	Fed vs Starvation	1.9182	0.170
	MG vs NMG	0.6889	0.409
	Fed vs Starvation × MG vs NMG	0.0367	0.849
Longevity (days)	Fed vs Starvation	19.7641	0.001
	MG vs NMG	1.4571	0.230
	Fed vs Starvation × MG vs NMG	0.0108	0.918

Data of these parameters didn't fit the normal distribution, thus the two-way ANOVA for trimmed means with interactions effects was applied, and Q-value and *p*-value were returned.

Table S9: The content of energy substances in migratory (MG) and non-migratory (NMG) individuals of *C. medinalis* under different nutritional conditions.

Treatment		Thorax		Abdomen	
		Triglyceride (mmol/gprot)	Glycogen (mg/mgprot)	Triglyceride (mmol/gprot)	Glycogen (mg/mgprot)
Fed	MG	0.0535 ± 0.00522	0.0071 ± 0.00051	0.1544 ± 0.02931	0.0389 ± 0.00450
	NMG	0.0522 ± 0.00248	0.0093 ± 0.00124	0.1368 ± 0.01456	0.0353 ± 0.00320
Starvarion	MG	0.0332 ± 0.00220	0.0103 ± 0.00082	0.1654 ± 0.01272	0.0361 ± 0.00087
	NMG	0.0361 ± 0.00632	0.0133 ± 0.00065	0.1320 ± 0.01156	0.0352 ± 0.00289

Data are presented as mean ± SD.

Table S10: Two-way ANOVA for the effects of nutritional conditions, migratory status on the content of thoracic and abdominal energy substances in migratory (MG) and non-migratory (NMG) individuals of *C. medinalis*.

Parameters	Tissue	Source	df	*F*	*p*-value
Triglyceride (mmol/gprot)	Thorax	Fed vs Starvation	1	50.863	＜0.001
		MG vs NMG	1	0.086	0.777
		Fed vs Starvation × MG vs NMG	1	0.662	0.439
		Error	8		
	Abdomen	Fed vs Starvation	1	0.062	0.809
		MG vs NMG	1	4.349	0.071
		Fed vs Starvation × MG vs NMG	1	0.421	0.535
		Error	8		
Glycogen (mg/mgprot)	Thorax	Fed vs Starvation	1	53.694	< 0.001
		MG vs NMG	1	28.2	0.001
		Fed vs Starvation × MG vs NMG	1	0.595	0.463
		Error	8		
	Abdomen	Fed vs Starvation	1	0.618	0.454
		MG vs NMG	1	1.555	0.248
		Fed vs Starvation × MG vs NMG	1	0.53	0.488
		Error	8		
